# Tooth brushing learning methods: time to change practice?

**DOI:** 10.1038/s41432-025-01175-y

**Published:** 2025-06-19

**Authors:** Patrick Quinn, Mairead Harding

**Affiliations:** Cork University Dental School and Hospital, University College Cork, Cork, Ireland

**Keywords:** Tooth brushing, Dental caries

## Abstract

**A Commentary on:**

**Leghrouz L, Khole M R, Splieth C H, Schmoekel J**.

Tooth Brushing Learning Methods: Differential or Conventional? – A Randomized Controlled Clinical Trial. *Caries Res* 2024; **58:** 399–406.

**Design:**

A two-arm single-blinded randomised controlled clinical trial to investigate the effects of the differential learning method versus conventional tooth brushing instruction by assessing changes in plaque levels and gingivitis in children aged three to eight years.

**Case Selection:**

Children aged three to eight years with poor oral hygiene at baseline, needing parental assistance with tooth brushing, and available to attend follow-up appointments were included in the study. Exclusion criteria included children with acute dental pain, those with serious systemic diseases requiring special attention during dental care, and those who refused to participate in the study. Participants were randomly assigned to the test and control groups by self-drawing an unlabelled envelope from a box. The sealed envelopes contained oral hygiene instructions with exercises to use the differential learning method for the test group and children in the control group received the usual tooth brushing instructions. Participants were asked to follow the instructions at home for 28 days.

**Data Analysis:**

The sample size was calculated according to previous similar oral hygiene studies, with a final sample size of 29 participants in each group to allow for dropouts of approximately 30%. Data was analysed using Microsoft Excel, with the significance threshold set at p < 0.05. Descriptive analysis included the calculation of means, standard deviation, absolute numbers, and percentages. Comparisons between the two groups were made using the independent samples *t*-test for quantitative variables and the chi-squared test for categorical variables.

**Results:**

Two calibrated and blinded examiners recorded the papillary bleeding index (PBI) and the Quigley-Hein Index for dental plaque (QHI) at baseline and at the first and second recall visits at four and twelve weeks respectively. Of the 58 children recruited for the study, 46 were included in the final analysis with 22 in the control group and 24 in the test group. At baseline, there were no significant differences between the groups with respect to plaque and gingival indices. At the first recall, a statistically significant difference in the PBI index was found in favour of the test group (test: 0.1 ± 0.2 v. control: 0.3 ± 0.2; p < 0.001) but the difference in relation to the QHI index was not statistically significant (test: 2.1 ± 0.9 v. control 2.6 ± 0.9; p = 0.07). At the second recall, statistically significant differences in both indices were found in favour of the test group (PBI test: 0.1 ± 0.2 v. PBI control: 0.5 ± 0.2; p < 0.001; QHI test: 2.1 ± 0.9 v. QHI control: 3.2 ± 1; p < 0.001).

**Conclusions:**

The authors of the study concluded that simple instructions with the differential learning method for home tooth brushing can lead to significantly greater improvements in oral hygiene in children with poor oral hygiene when compared to traditional instructions, particularly in the medium term retention phase.

## GRADE Rating:



## Commentary

This study by Leghrouz et al. examines the role of differential learning in tooth brushing, an emerging approach in oral health education^1^. Traditional tooth brushing instruction often involves a motor learning approach based on repetition and corrective instruction to achieve competence^2^. Differential learning involves maximal variation of the movement to stimulate the learner to find their optimal performance^2^. For example, in the study by Leghrouz et al., participants in the test group were asked to perform tooth brushing with nine different exercises such as while lying down, with the non-dominant hand, and with closed eyes^1^. Differential learning has been successfully applied to the training of sport techniques and handwriting, and recent studies are beginning to explore a possible role in tooth brushing education^2^.

The study by Leghrouz et al. has many strengths^1^. The CONSORT checklist and the RoB-2 risk of bias tool were utilised in the study conception phase to assure quality and minimise bias. Sample size calculations were undertaken based on previous similar studies, a process of randomisation was put in place to assign participants to groups, and investigators were blinded as regards the participant groups. Investigators were trained and calibrated in the indices used in the study, with very good agreement being achieved for both inter-examiner and intra-examiner weighted kappa scores. Finally, the distribution of dropouts was analysed and dropout-related bias was found to be unlikely.

However, it is suggested that there are a number of issues relating to this study that the reader should take into consideration when interpreting and applying the findings. The authors acknowledge that, as children of this age are usually assisted by their parents while brushing, parental involvement should be taken into account when interpreting results. Following on from this, the exclusion criteria did not include factors that might impact on tooth brushing ability, such as children or parents with impaired manual dexterity. Unlike a similar study by Pabel et al.^2^, the present study did not standardise the type of toothbrush and toothpaste used across groups, although the authors report no statistically significant differences regarding the type of brush used. It is also important to highlight that compliance was assessed by asking the participants to place a sticker on a sheet for every day they brushed according to instructions. There is a possibility of misreporting of compliance with this method, and in the present study only 13 of the 22 participants in the control group and 18 of the 24 participants in the test group filled and returned the sticker sheet.

The authors acknowledge the possible impact that the Hawthorn effect may have had on the findings. As the reader will know, the Hawthorn effect is a phenomenon whereby research participants may change their behaviour simply because of the fact that they are being observed^3^. In the present study, it is possible that both groups may have altered their brushing behaviour for this reason. However, while participants in both groups showed significant improvements in oral hygiene at the first recall, the results were preserved at the second recall only for the differential learning (test) group. The authors also mention that the COVID-19 pandemic resulted in delays to the planned schedule for the first and second follow-up appointments, with mean values for the second follow-up of 19.4 + /- 7 weeks in the control group and 20.1 + /- 8.1 weeks in the test group. The variability in follow-up periods needs to be taken into consideration when interpreting findings, although the authors suggest that a longer follow-up time possibly led to clearer conclusions.

This research builds on the recommendations of previous research by Pabel et al. that differential learning should be studied in children with poor oral hygiene^2^. While it is suggested that there are a number of issues that should be taken into consideration when interpreting the results, the study by Leghrouz et al. concludes that differential learning may have a role in improving oral hygiene in the medium term. As is so often the case, further research is needed, such as on the effectiveness of differential learning in the longer term and on the impact of the technique on actual disease levels. The practicality of the technique will also need consideration, as ideally it should easily assimilate into day-to-day living. In conclusion, while it may not yet be time to change how we practice tooth brushing instruction, the differential learning approach merits further exploration and may in time play a role in oral health education.

Practice Points

- This randomised controlled clinical trial suggests that differential learning was superior to conventional learning methods in improving oral hygiene in children with poor oral hygiene and high caries experience in the medium term. However, there are a number of issues relating to the research that should be taken into consideration when interpreting and/or applying the results.
- Further research is needed in areas such as the effectiveness of differential learning in the longer term and the impact of the approach on disease levels. The practicality of the approach in everyday living will also need consideration.
